# Xeroderma Pigmentosum C: A Valuable Tool to Decipher the Signaling Pathways in Skin Cancers

**DOI:** 10.1155/2021/6689403

**Published:** 2021-04-28

**Authors:** A. Nasrallah, N. Fayyad, F. Kobaisi, B. Badran, H. Fayyad-Kazan, M. Fayyad-Kazan, M. Sève, W. Rachidi

**Affiliations:** ^1^Univ. Grenoble Alpes, SYMMES/CIBEST UMR 5819 UGA-CNRS-CEA, IRIG/CEA-Grenoble, Grenoble, France; ^2^Univ. Grenoble Alpes, CEA, Inserm, BIG-BGE U1038, 38000 Grenoble, France; ^3^Laboratory of Cancer Biology and Molecular Immunology, Faculty of Sciences I, Lebanese University, Hadath, Lebanon; ^4^Institut de Biologie et Pathologie, PROMETHEE Proteomic Platform, CHU Grenoble Alpes, 38000 Grenoble, France

## Abstract

Xeroderma pigmentosum (XP) is a rare autosomal genodermatosis that manifests clinically with pronounced sensitivity to ultraviolet (UV) radiation and the high probability of the occurrence of different skin cancer types in XP patients. XP is mainly caused by mutations in XP-genes that are involved in the nucleotide excision repair (NER) pathway that functions in the removal of bulky DNA adducts. Besides, the aggregation of DNA lesions is a life-threatening event that might be a key for developing various mutations facilitating cancer appearance. One of the key players of NER is XPC that senses helical distortions found in damaged DNA. The majority of XPC gene mutations are nonsense, and some are missense leading either to the loss of XPC protein or to the expression of a truncated nonfunctional version. Given that no cure is yet available, XPC patients should be completely protected and isolated from all types of UV radiations (UVR). Although it is still poorly understood, the characterization of the proteomic signature of an XPC mutant is essential to identify mediators that could be targeted to prevent cancer development in XPC patients. Unraveling this proteomic signature is fundamental to decipher the signaling pathways affected by the loss of XPC expression following exposure to UVB radiation. In this review, we will focus on the signaling pathways disrupted in skin cancer, pathways modulating NER's function, including XPC, to disclose signaling pathways associated with XPC loss and skin cancer occurrence.

## 1. Introduction

All living organisms are ceaselessly under the risk of exposure to various agents interfering with their DNA, RNA, and protein integrity [1]. The durability of cells, tissues, and organs depends roughly on DNA's stability. Genomic attacks are copious due to exogenous environmental factors ranging from physical factors like ionizing radiation (IR), ultraviolet (UV) radiation, to harmful chemical agents as well as endogenous factors. Such agents interfere with the chemical composition of the DNA double helix by creating helical distortions considered signs of lesions. Altogether, harmful burdens may induce up to 10^4^–10^5^ DNA lesions per mammalian cell per day [2]. Solar ultraviolet radiation (UVR) is a physical electromagnetic radiation that is considered a DNA-damaging agent. It is divided into 3 categories based on the wavelengths: UVA (320−400 nm), UVB (280−320 nm), and UVC (100−280 nm) [3, 4]. When DNA absorbs UVB or UVC radiations, two primary photoproducts are generated: 6-4-pyrimidine-pyrimidone photoproducts [(6-4) PPs], and cyclobutane pyrimidine dimers (CPDs). Upon lesion sensation, repair systems trigger a cascade of events that leads to the detection, correction, and restoration of the initial genetic information and preventing cancer development [5, 6]. Several DNA repairing systems exist: the nucleotide excision repair (NER), base excision repair (BER), direct reversal repair, double-stranded break repair (DSB), and interstrand crosslink repair (ICL) [7]. One of the most critical pathways involved in the removal of bulky DNA adducts caused by UV light is the nucleotide excision repair (NER) pathway [8]. NER pathway deficiencies lead to the development of various genetic disorders, including xeroderma pigmentosum (XP), the main focus in our review. XP is a rare autosomal recessive genodermatosis varying in terms of nomenclature from (XP-A to XP-G and XP-V) depending on the type of mutation affecting one of eight different *XP* genes [9–11]. XPC has the most significant number of patients, with 80 to 90% of cases, depending on the country of origin. However, an ongoing debate about the heterogeneity of XP-C patients in terms of the clinical manifestations (nonmelanoma and/or melanoma skin cancers) does exist. The *XPC* gene is located on chromosome 3p25, comprising about 15 introns and 16 exons encoding for a functional XPC protein [12]. XPC is a DNA repair protein involved in DNA damage recognition in the NER pathway allowing further events to occur, restoring the standard DNA copy. Furthermore, the XPC protein might also be involved in vital parts of DNA damage responses, including programmed cell death and cell cycle checkpoints [19]. Upon the loss of XPC's expression, and after extensive UVB-induced DNA lesions, mutations might develop in genes encoding for essential proteins involved in the signal transduction pathways. The latter is essential to convey external stimuli into the target cell. Disorganization of signal transduction pathways is a major cause of skin cancer development in XPC patients. The aim of our work is to characterize the proteomic signature of XPC at basal state and after UVB irradiation. In this article, we will review signaling pathways disrupted in skin cancer to understand how skin cancers develop in XPC patients. Furthermore, we will also discuss about signaling pathways affecting NER's activity, proteins linked to signaling pathways influencing wild-type XPC's function, and a little information about XPC loss and skin cancer.

## 2. Signaling Pathways Disrupted in Skin Cancer

Skin cancer is one of the most common cancer types occurring in the overall population of people, especially in the white populace; with over a million cases distinguished in the world every year [13–15]. Skin cancer classification and nomenclature depend on the type of cells from which they arise and the clinical outcome. The two most common subtypes of nonmelanoma skin cancer (NMSC) are basal cell carcinomas (BCCs) and cutaneous squamous cell carcinomas (SSCs) both originating from the basal layer of the skin epidermis [16]. Although they share many similarities, they have different incidence rates and etiology. BCCs are considered to be the most frequent (80%-85%) followed by SCCs (15-20%). BCC is slow-growing and rarely metastatic (less than 0.01%), and most of them are often treated by surgery. Even though mortality is low, this threat causes morbidity and a colossal weight on social insurance frameworks around the world [17–19]. On the other hand, the prognosis of SCCs is worse because they can be invasive with a significant propensity to metastasize (2-5%). Besides, a high percentage of SCC patients develop second primary skin cancer within 5 years of diagnosis. The third main class of skin cancer, malignant melanoma, originating from the melanocytes, is less frequent (less than 10%) but is associated with higher morbidity and mortality (15-20%) than NMSC [13, 20]. Two significant hazard factors that are related to the pathophysiology of numerous cutaneous carcinogenesis are environmental (likewise named modifiable) and hereditary (additionally named nonmodifiable) factors [21, 22]. The most well-known environmental hazard factor for all skin malignancy types is the exposure to UVR, which can damage DNA in skin cells like keratinocytes, melanocytes, and fibroblasts [17, 18]. In multicellular life forms, the regulation of gene expression is attained via signaling transduction pathways, mediating the development of well-organized physiological processes fundamentally engaged with skin cells development, proliferation, division, death, and differentiation [23, 24]. Understanding the intracellular signals as well as the processes through which cells receive and incorporate extracellular stimulus is critical for the identification and advancement of novel therapeutics for cutaneous malignancies. The mechanism of UVR at the molecular level is related with the expanding various DNA harm signals, e.g., initiation of the p53 pathway which significantly adjusts cell physiology to intercede cell cycle arrest and enact DNA repair. If DNA remains unrepaired, p53 can directly trigger programmed cell death to prevent tumor development [25, 26]. Strikingly, the exposure of human keratinocytes to UVB radiation initiates the PI3K/AKT/mTOR-S6K1 pathway [27]. The latter is a key pathway engaged with an assortment of physiologic capacities linking nutrients and growth factors to metabolism, cell development, proliferation, angiogenesis, survival, apoptosis, and protein and lipid production. This pathway is dysregulated in various malignancies including melanoma and nonmelanoma skin tumors [28, 29]. In this part, we will discuss the molecular signaling in NMSC and cutaneous melanoma (CM).

### 2.1. Signaling Pathways in NMSC

**Figure 1:**
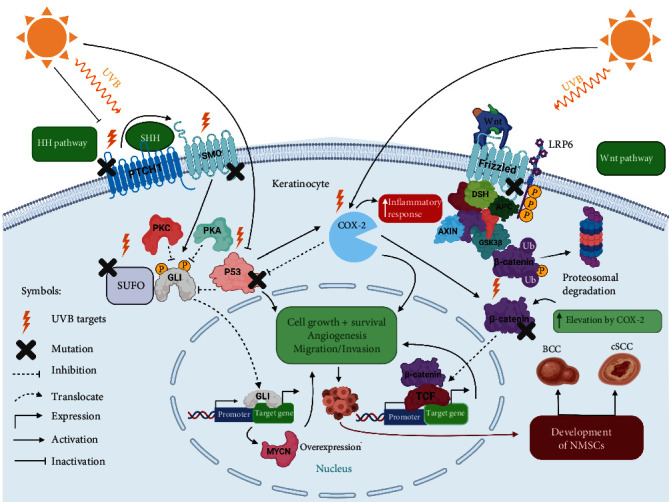
Development of NMSCs due to the dysregulation of Wnt, HH, p53, and COX-2 signaling pathways in keratinocytes triggered by UVB radiation. UVB radiation can cause alterations in PTCH1, SMO, and SUFO in the HH pathway and p53 and *β*-catenin in Wnt pathway that will eventually drive constitutive expression of downstream effectors which are GLI1, *β*-catenin, and COX-2 favoring the overexpression of target genes (protooncogenes) like N-MYC leading to uncontrolled cell growth, survival, angiogenesis, and migration and invasion required for NMSCs (BCC, SCCs). “Created with http://BioRender.com/.”

Basal cell carcinoma (BCC) and cutaneous squamous cell carcinoma (SSCs) are derived from dysregulated keratinocytes present in the basal layer of the epidermis, both establishing the principle types of nonmelanoma skin cancer (NMSC) [30, 31]. The morphological highlights of BCC comprise the appearance of a gathering of tumors that are made out of cells having cellular constituents like basal epidermal cells in the undifferentiated state. A significant component of BCC is the palisade course of action of epidermal cells in the tumor fringe that isolates the tumor from the encompassing stroma. These cells regularly give the tumor nodular shape or structure a band or string encompassing it. Contrasted with their typical partners, the tumor cells have less chromatin-rich nucleus and cytoplasm, favoring mitotic division and also apoptotic cell demise, reflecting steady development of the tumor. Clinical manifestation of BCC may show up in various morphological examples: nodular or cystic, shallow, infiltering, and sclerotic or pigmented, which also differ in their site event [32, 33]. The second most common type of NMSC is generally SSCs [34]. SSCs can metastasize from the epidermis to the dermal layers as well as to the local lymph nodes where around 5% of patients developed metastatic tumors [35]. Clinical introduction including however not constrained to hyperkeratotic plaque also leads to the arrangement of nodular mass or ulceration on the skin, which might be related to torment, pruritus, or blood draining [36]. Actinic keratosis (AK) and Bowen's sickness are two premalignant types of SSCs, which, if not treated well, would develop malignant transformation phenotype [37]. Even though most of the SSCs are effectively removed by surgical intervention, around 20% of skin cancer deaths are linked to SSCs [38]. Given its expanding frequency and poor prognosis, SSCs is emerging as a public medical problem. Understanding the molecular pathways relying behind skin cancer development as a result of UVR exposure is quite complicated. UVR can be classified as a carcinogen capable of initiating and promoting skin cancer due to its capacity to reach the basal layer of the epidermis, triggering alterations in keratinocytes. Initiation of skin tumors in response to UVR exposure can occur through the activation of signaling pathways that favor the survival stimulus in keratinocytes, opposing the programmed cell death pathway. Activation of these signaling pathways can occur through direct DNA damage of critical genes that might act either as oncogenes or tumor suppressor genes, activation of transmembrane receptors involved in signal transduction cascade events like receptor tyrosine kinases (RTKs), or via the elevation of inflammatory responses and immunosuppression mechanisms [39, 40]. The upregulation of these pathways can then promote carcinogenesis by favoring the proliferation of these damaged cells [41]. Understanding the dysregulation of the signaling pathways at the molecular level as a result of UVB exposure would pave the way towards specific targeted therapy to treat NMSC. Upregulation of the *Hedgehog* pathway has been shown as an essential component required for NMSC progression; however, other nonstandard pathways, for example, *Wnt/β-catenin*, *p53*, *p16*, *COX-2*, *CDKI2A*, *PI3K/AKT/mTOR-S6K1*, and *Ras-Raf-MEK-ERK* signaling pathways have been also involved in the pathogenesis of NMSC (Figure 1).

#### 2.1.1. Hedgehog Signaling Pathway

Sonic Hedgehog (SHH), a highly conserved pathway, is associated with organogenesis, development patterning, tissue growth, mitogenesis, homeostasis, tissue repair, and hair follicle growth and sebaceous glands in the skin [42]. The canonical HH pathway comprises the HH ligands as Indian HH, Sonic HH, and desert HH, the transmembrane receptor proteins patched (PTCH1 and PTCH2) which are classified as tumor suppressor genes, smoothened (SMO), and glioma-associated oncogene (GLI) transcription factors 1, 2, and 3 (GLI1, GLI2, and GLI3) [43]. This pathway is enacted when HH ligands bind to and inhibit PTCH1, thereby initiating SMO downstream effects which in the absence of HH ligands is repressed by PTCH1 [44]. SMO translocates and accumulates in the primary cilium where this pathway befalls, allowing further cascade events to occur which in turn prompts the departure of the GLI proteins, sequestered in the cytoplasm with various proteins negatively controlling them like p53, PKA, and PKC-*δ* and the suppressor of fused (SUFU). Then, GLI proteins enter into the nucleus and turn on the expression of GLI-associated genes, which are responsible of producing proteins responsible for the cellular destiny, associated with proliferation, viability, angiogenesis, and self-renewal. GLI1 exerts a negative feedback loop that autoregulates HH signaling by modulating PTCH1 [45]. UVR induced mutations at any level of the HH signaling pathway (e.g., PTCH1, SMO, and SUFU) will result in an increased expression of GLI1 leading to constitutive proliferation that favors skin tumorigenesis. The SHH pathway is mostly associated with the etiology of BCC. Furthermore, mutations occurring in the *PTCH1* gene have been characterized in BCC patients with rare genetic disorders including xeroderma pigmentosum (XP) as well as in sporadic BCC. Around 60% of BCC and XP patients have shown that most of *PTCH* point mutations acquire the UV signature (i.e., C → T and CC → TT transitions at dipyrimidine sites) [46]. Besides, UVR induced mutations in the *PTCH1* gene account for around 50% of sporadic BCCs where large and small deletions occur within this gene, leading to uncontrolled cell cycle progression, inhibition of apoptosis, induction of angiogenesis, and proliferation which are hallmarks for NMSC [47]. Furthermore, it was also shown that SSCs has the potential to overexpress Sonic Hedgehog (SHH), PTCH, and the most important target of HH signaling GLI1 [48, 49]. In addition, mouse models having SSCs have also shown that PTCH1 had the potential to act as an oncogene, if overexpressed [50]. These alterations inactivate PTCH's work and permit constitutive actuation of the SMO-GLI pathway, which is by all accounts adequate for NMSC advancement [45]. UVR can also cause mutations in *SMO* and *SUFO* genes causing aberration in the HH signaling pathway [51].

#### 2.1.2. Wnt/*β*-Catenin Signaling Pathway

The Wnt (Wingless/INT-1) signaling is another pathway playing vital roles in cellular expansion and proliferation as well as hair growth and development in the skin. Similar to the HH pathway, those two pathways crosstalk to sustain normal physiological processes in the human body. Besides, dysregulation of the Wnt pathway is implicated in the development of various cancers including NMSC [52]. *β*-catenin is a vital actor mediating the downstream effects of the Wnt signaling pathway. *β*-catenin is usually implicated in the formation of adherens junction between cells found in the skin [53]. In the absence of Wnt, *β*-catenin is sequestered in the cytoplasm by various protein regulators including adenomatous polyposis coli (APC), axin, and glycogen synthase kinase *β* (GSK3*β*), where *β*-catenin is phosphorylated by GSK3*β* and targeted to proteasomal degradation to ensure its control and regulation. When Wnt is coupled to its receptors, LDL receptor-related protein (LRP-6), and frizzled belonging to a family of 7-transmembrane receptors at the surface of the cell membrane, GSK3*β* becomes inactivated. This causes *β*-catenin to remain dephosphorylated, thus preventing its proteasomal degradation. *β*-catenin can thereafter translocate to the nucleus, where it exerts its function as a transcriptional coactivator binding to TCF (T cell factor) motif where TCF then binds and turns on the expression of Wnt target genes. Obviously, the Wnt signaling pathway appears to act as a key controller in NMSC's progression. Various genomic and transcriptomic investigations have revealed that the Wnt pathway is being dysregulated in SCCs. Perhaps the most punctual examination utilizing genomic hybridization discovered amplification of chromosome arms 7q, 8q, 11q, and 17q which includes Wnt and frizzled genes in SCC lines suggesting its link with SCC progression [54]. Another study showed the increase of Wnt ligands and receptors at the mRNA level using gene expression array analyses in SCC samples [55]. Mutations in APC which is classified as a tumor suppressor gene will eventually lead to the destabilization of the complex sequestering *β*-catenin allowing its constitutive translocation to the nucleus. In addition, mutations of *β*-catenin or mutations in axin would all lead to the constitutive activation of the Wnt signaling pathway, thereby driving extensive expression of *Wnt* target genes which favors the development of BCCs [56]. Increased expression of Wnt proteins has also been shown as a significant activator of this pathway observed in BCCs [57, 58]. Dysregulation of the *Wnt/β-catenin* signaling favors the upregulation of *BIRC5/Survivin* which is implicated in inhibiting caspases mainly caspase-3 and caspase-7 thus preventing apoptosis leading to the immortality of tumor cells [59]. Also, UVB can be an important cause for the rise of *β*-catenin levels acting on cyclooxygenase-2 (COX-2) thus augmenting the production of prostaglandin E2 (PGE2) which in turn increases the inflammatory response that favors *β*-catenin's elevation thereby leading to the constitutive activation of this pathway allowing the development of skin carcinogenesis [60].

#### 2.1.3. p53, p16, and COX-2 Signaling Pathways

At any time, the cell has several systems for arresting the cell cycle if the cascade of events does not occur in an orderly fashion, like when the DNA is damaged. The *TP53* gene is classified as a key tumor suppressor gene playing vital roles in cell cycle regulation. p53 is a proapoptotic protein, belonging to the Bcl-2 protein family that guards the genome to ensure its integrity and stability by orchestrating DNA damage responses [61]. Exposure to mutagenic environmental factors would eventually create DNA lesions sensed by p53, which activates cell cycle checkpoints to initiate DNA repair. If repair systems were incapable of resolving the error, p53 can halt the cell cycle events and trigger programmed cell death in order to prevent the development of tumors. Extensive exposure to UVB radiation absorbed by the keratinocyte's DNA will eventually cause mutations in the *TP53* gene, leading to its inactivation thus favoring the development of skin cancers. *TP53* gene mutation can be present in around 50% of BCC and approximately 90% of SCC cases [128]. Biopsy analysis revealed that sun-exposed skin showed C → T transversions in DNA sequences of *TP53* named as p53 patches [62]. The recurrence of C to T transitions most commonly occur at the trinucleotide sequence 5′-PyCG in the *TP53* gene in UV-induced skin tumor [63]. Hotspot mutation sites present in the *TP53* gene are numerous caused mainly by UVB radiation. UVB lamps inducing skin tumors in mice have identified a hotspot mutation at codon 270 correlated to a sequence change from 5′-TCGT to 5′-TTGT [64]. Furthermore, mutations in codon 177 of the *TP53* gene are classified as a specific marker for BCC development, whereas mutations in codon 278 appear to be explicit for SCCs [50]. The expression level of p53 can be used as a prognostic marker for various skin cancers. p16 is another important key protein encoded by the *CDKN2A* gene which is a tumor suppressor gene regulating negatively the cell cycle progression in the G1-S transition by inhibiting cyclin-dependent kinase 4 (CDK4). Mutations in the *CDK2NA* gene would drive the cell cycle progression thereby allowing the constitutive proliferation of skin cells and the development of skin tumors. UVR is associated with causing alterations within this gene. Exon 2 of the *CDK2NA* gene had six distinct mutations: 5 out of 21 patients suffering from squamous lesions and 1 out of 28 BCCs patients. The UVR signature presented in exon 2 of this gene included two C : G to T : A transitions and two transversions [65]. Cyclooxygenase-2 (COX-2) is a key enzyme that catalyzes the conversion of arachidonic acid to prostaglandins playing an important role in increasing the inflammatory responses [66]. The major product produced upon COX-2 activation is the prostaglandin E2 (PGE2) which is highly synthesized after UV exposure due to COX-2 activity [67]. This prostaglandin exerts various effects at the biological level by suppressing programmed cell death and enhancing cellular proliferation [68]. There is a strong correlation between the overexpression of COX-2 and NMSC development. A major cause of COX-2 overexpression could be due to UV-B radiation exposure and the dysregulation of various signaling pathways through their permanent activation, like PI3K, MAPK, and NF-*κ*B, that drives uncontrolled cellular expansion and proliferation. Under normal physiological conditions, wild-type p53 has a negative impact on COX-2 by decreasing its expression. Besides, there was no significant reduction in the COX-2 levels in p53 mutant [69]. This could be a major reason for understanding why COX-2 levels are high in various cancers including BCC and SCCs and low in normal epithelial cells including keratinocytes. Upon their mutation, p53 and p16 comprise two unique pathways that could turn on the COX-2 activity to increase PGE2 levels favoring NMSCs.

#### 2.1.4. PI3K/AKT/mTOR Signaling Pathway

**Figure 2:**
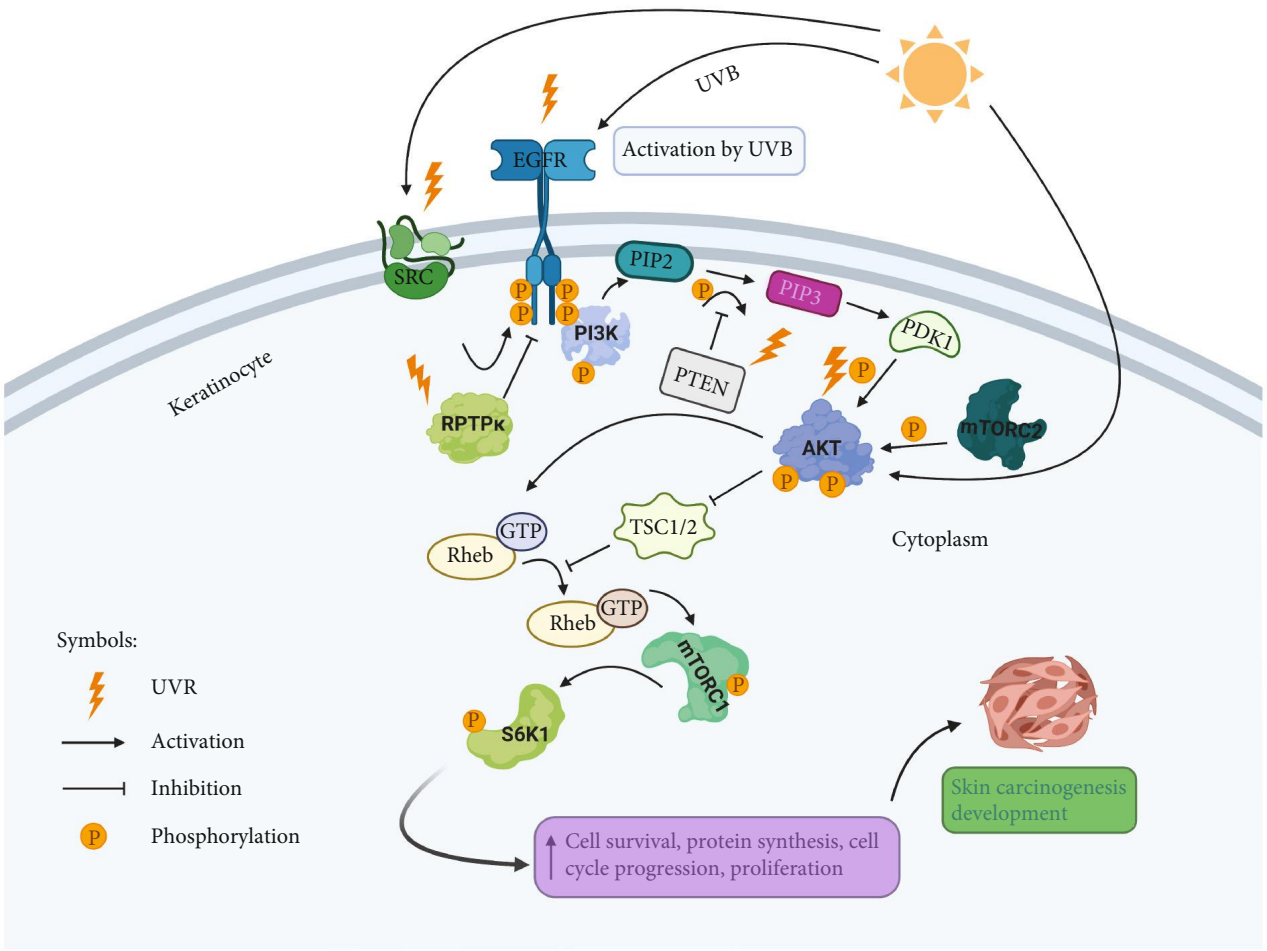
The key targets of UVR in the *PI3K/AKT/mTOR-S6K1* pathway. UVB exposure can cause various alterations in the *PI3K/AKT/mTOR-S6K1* pathway where any functional loss of PTEN or RPTP-*κ* or hyperactivity of EGFR, Src, or AKT would eventually lead to constitutive cell survival, protein synthesis, cell cycle progression, and proliferation, which are the hallmarks of skin carcinogenesis. “Created with http://BioRender.com/.”

Transmembrane receptors are embedded in the cell membrane, participating in various biological processes. One huge family of such receptors is receptor tyrosine kinases (RTKs). Epidermal growth factor receptor (EGFR) is categorized as an RTK. The standard pathway begins upon the coupling of epidermal growth factor (EGF) on its receptor inducing receptor dimerization and phosphorylation of tyrosine residues in the cytosolic portion of the receptor to mediate downstream effects via intracellular signaling cascades. The upregulation of EGFR has been reported in SCCs [70]. Skin tumorigenesis requires EGFR activation in keratinocytes caused by UVR [71]. The generation of reactive oxygen species (ROS) due to UVR exposure could be responsible for the rapid activation of EGFR rather than through growth factor binding [72]. An important regulator of the EGFR activity is receptor-type protein tyrosine phosphatase- kappa (RPTP-*κ*), maintaining EGFR in its inactive state, unphosphorylated. Under UVR exposure conditions, ROS triggers oxidative inhibition of RPTP-*κ* thus leaving EGFR uncontrolled [73]. Protooncogene tyrosine-protein kinase Src may be also activated by UVR thus phosphorylating EGFR promoting carcinogenesis [74]. EGFR activated via UVR stimulates various downstream effectors, including the *PI3K/AKT/mTOR-S6K1* pathway. Initiation of this pathway occurs upon the activation of RTKs and EGFR leads to the actuation of phosphatidylinositol 3-kinase (PI3K), which in turn phosphorylates phosphatidylinositol [4, 5]-bisphosphate PIP2 to phosphatidylinositol [3, 4, 5]-trisphosphate PIP3. In the activation of protein kinase B (AKT), a serine/threonine-specific protein kinase occurs through the binding of PIP3 to the N-terminus of AKT, allowing its translocation to the inner leaflet of the plasma membrane where it becomes phosphorylated by phosphoinositide-dependent kinase-1 (PDK1), and by the mammalian target of rapamycin (mTORC2). Regulation of AKT's function occurs via phosphatase and tensin homolog deleted from chromosome ten (PTEN) which is classified as a tumor suppressor gene. AKT is an essential part of this pathway required to convey the signals that are responsible for the regulation of various cellular processes. Furthermore, AKT then activates mTORC1 through Rheb-GTP. mTORC1 phosphorylates downstream p70S6 kinase 1 (S6K1) involved in protein synthesis, cell cycle progression, cell growth, and survival (Figure 2). According to recent studies, UVR can regulate PTEN's function in keratinocytes [75]. Exposure to UVR causes modifications in the *PTEN* gene [76]. UVR also can inhibit PTEN's function through ROS, favoring AKT activation thus the development of skin tumors [77]. At the level of BCC, even though little is known with regard to PTEN's function in BCC, upregulation of the *PI3K/AKT* pathway could be due to *PTEN* gene mutation [78]. Decreased PTEN expression levels lead to a threatening change of the skin actuated by UVA (315–400 nm), as shown by the development of SCC in nude mice [75]. UVR-induced AKT actuation can occur via an autocrine manner or ROS activating growth factor receptors bearing RTK activity [79]. It is worth mentioning that the *PI3K/AKT* signaling pathway also affects other signaling pathways including *Ras-Raf-MEK-ERK* which due to the crosstalk presence could also be dysregulated favoring skin tumorigenesis.

### 2.2. Signaling Pathways in Cutaneous Melanoma

The overall rate of cutaneous melanoma (CM) has been increasing every year at a progressively rapid rate contrasted with some other types of skin cancers [80]. CM originates from the transformation of melanocytes which are derived from neural crest cells located mainly in the epidermis of the skin [81]. Although it is the least common form comprising around 1% of total skin cancers, CM is classified as the most aggressive form due to its high metastatic capacity [82]. The occurrence of cutaneous melanoma varies enormously among nations, and these diverse frequency designs are attributed to varieties in skin phototype, just as contrasts in sun exposure. Additionally, and in contrast to other solid tumors, melanoma generally influences youthful and moderately aged people (middle age at analysis, 57 years). The frequency increments directly after the age of 25 years until the age of 50 years, and afterward, starts decreasing, especially in females [83]. The major cause of CM development relies on the degree of sun's exposure where UV-B radiation is the most implicated factor for inducing melanomas as an environmental factor where it could be direct when melanocyte's DNA absorbs UVB photons or indirect in which fluorophores including flavins and porphyrins absorb UVB photons and generate ROS that encompasses mainly hydrogen peroxides and superoxide anions leading to the disruption of signaling pathways at the molecular level [84]. CPDs resulting from UV-B radiation are the most common types of DNA lesions. TT > TC > CT > CC is the order of their formation from the highest (TT) to lowest (CC) frequency [85]. Another cause related to CM could be present at the genetic level as alterations in B-RAF were detected in ~60% while for N-RAS reveals ~15-30% of total melanomas [86]. Both of them are classified as main oncogenes essential for the mitogen-activated protein kinase (MAPK) pathway and mutations within these oncogenes would turn them on constitutively, dysregulating the MAPK pathway, thus favoring melanomas. Besides, B-RAF mutations were predominantly found in body areas harmed by the sun but at irregular intervals while for N-RAS they were present in damaged body areas that were continuously exposed to the sun [87, 88]. Codon 600 present within B-RAF's gene appears to be the most commonly mutated site where a single-base substitution allows the conversion of valine to glutamic acid producing a constitutively active protein version. In addition, the N-RAS gene also appears to have mutations at codon 61 converting glutamine to lysine [88]. Furthermore, the involvement of UV signature mutation at these sites is still unknown and not fully understood. A third newly discovered oncogene as being linked to cutaneous melanomas is RAC1. The latter is linked to the *PI3K/AKT* pathway where any activating mutation in RAC1 would dysregulate this pathway and favor cutaneous melanoma development. The most common mutation involves codon 29 converting proline to serine amino acid [89]. This mutation harbors a UV-signature mutation which is C > T transition where UVR is most probably responsible for this mutation [90]. Patients suffering from xeroderma pigmentosum or familial retinoblastoma disorders are at higher risk for developing CM. Dysregulation of signaling pathways in CM had been widely reported where the main signaling pathways include *PI3K*/*AKT*/*mTOR*, *Ras-Raf-MEK-ERK*, and *TGF-β* signaling pathways augmenting the aggressiveness of CM. *BRAF*, *NRAS*, *CDKN2A*, *CDK4*, and other RTKs like *ROS1*, *ALK*, *MET*, *RET*, and *NTRK1* are also other candidates for melanoma development to be considered [91, 92].

## 3. Signaling Pathways Modulating NER

**Figure 3:**
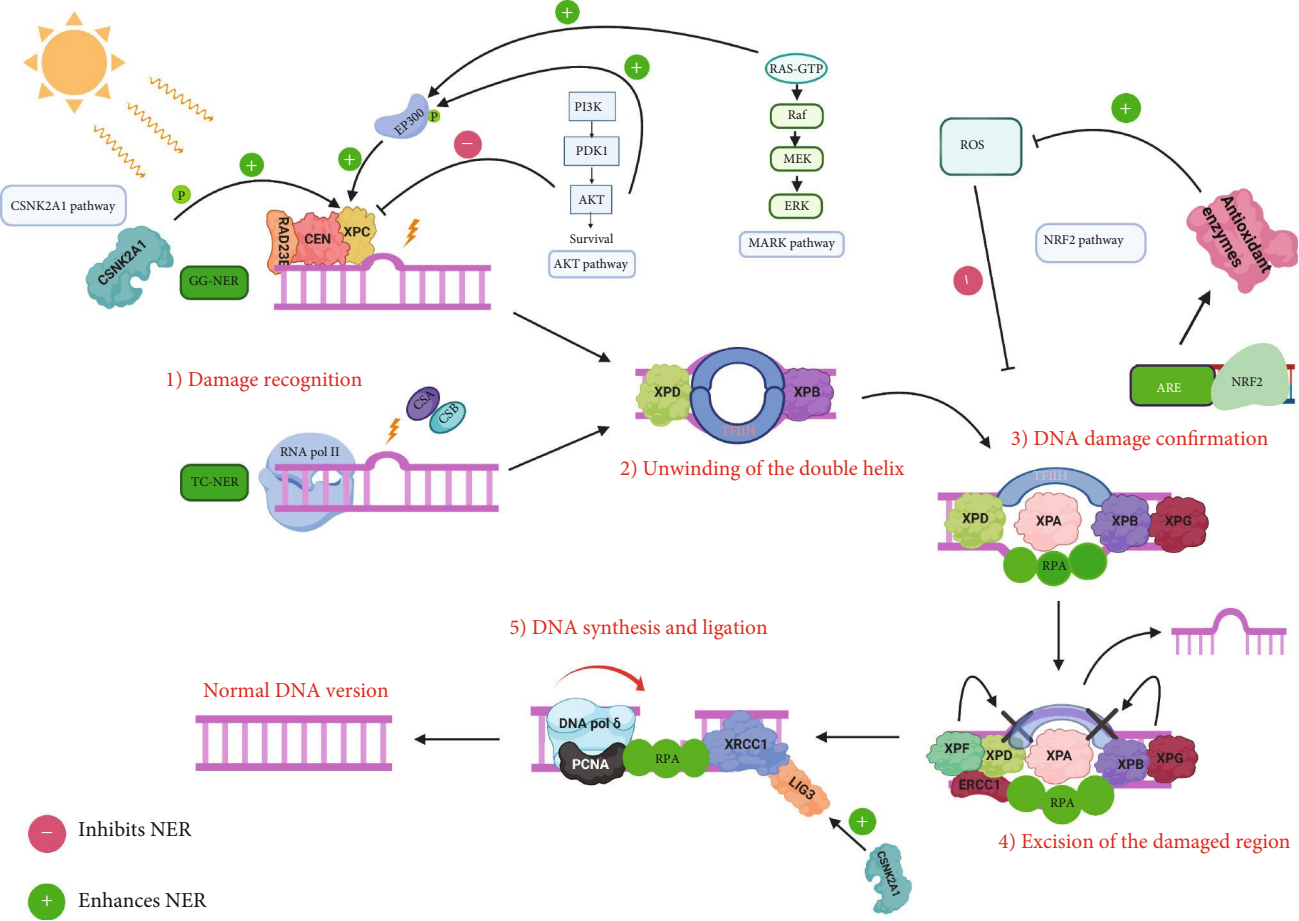
*PI3K/AKT1*, *CSNK2A1* (*CK2 α 1*), *MAPK*, and *NFE2L2* (*NRF2*) signaling pathways regulating NER's pathway. The *PI3K/AKT1* and *MAPK* pathways regulate XPC positively via EP300, the CSNK2A1 pathway also can affect positively both XPC and XRCC1-Lig3 complex, and the NRF2 pathway can enhance NER's activity positively by inducing the expression of antioxidant enzymes inhibiting ROS. On the other hand, AKT also can inhibit XPC under certain conditions. “Created with http://BioRender.com/.”

Nucleotide excision repair (NER) is a DNA repair pathway involved in the removal of bulky DNA adducts caused by exposure to UV light. NER can be divided into 2 subpathways: global-genome nucleotide excision repair (GG-NER) and transcription-coupled nucleotide excision repair (TC-NER) differing in the strategy of DNA damage recognition. Upon UVB exposure, damage recognition in the GG-NER occurs via XPC and other coupled key players like Rad 23 homologue B (RAD23B) and centrin 2 (CEN), while for TC-NER, this occurs via RNA polymerase II present in the expressed regions allowing the further recruitment of cockayne syndrome proteins A and B (CSA and CSB). The upcoming steps are similar for both subpathways where the second step involves the opening of the DNA double helix where transcription factor II H (TFIIH) participates and XPD and XPB helicases progress to unwind the DNA before repair. The third step involves DNA damage confirmation via replication protein (RPA1), XPA, and XPG. RPA1 binds to the single-stranded DNA preventing its rewinding and XPA confirms the damage. Upon verification, the fourth step which is excision involves the recruitment of XPF and excision repair cross-complementation group 1(ERCC1). Excision at the 5′ and 3′ sites of the damaged part of the DNA occurs via nucleases which are XPF and XPG. Finally, the fifth step involves DNA synthesis and ligation. DNA polymerase delta (Pol*δ*) with other important factors like proliferating cell nuclear antigen (PCNA) polymerize new complementary bases; then after synthesis, the ligation step is required to seal the gaps between nucleotides by DNA ligase 3 (LIG3) cooperating with X-ray repair cross-complementing protein 1 (XRCC1) which acts as a linker between Pol*δ* and LIG3 bringing them into close proximity ending up with the restoration of the normal DNA version [93]. NER's function can be modulated and influenced by various signaling pathways that might impact its repair activity in terms of removing DNA adducts either positively or negatively. PI3K/AKT1, CSNK2A1 (CK21), MAPK, and NFE2L2 (NRF2) are the main signaling pathways implicated (Figure 3).

### 3.1. PI3K/AKT1 Signaling Pathway Regulating NER

As mentioned previously, this pathway is switched on via the RTKs mediating the activation of PI3K1 which in turn activates several signaling cascades to activate AKT1. The inclusion of this pathway in the regulation of NER's work is yet disputable. AKT can activate the mouse double minute 2 homolog (MDM2) which is a negative regulator of p53 thus favoring its downregulation [94]. Along these lines, AKT1 has the potential to inhibit NER because of the reduction in the levels of XPC and damage specific DNA binding protein 2 (DDB2) which are dependent on TP53 for their expression at the transcription level and are considered key effectors in the NER pathway [95]. Another method of hindrance is interceded by the AKT1-dependent subcellular localization of XPC transcriptional repressors (P130) thereby inhibiting NER [96]. Besides, AKT1 can boost TC-NER through the phosphorylation of EP300 which acts as a histone acetyltransferase that undergoes chromatin remodeling processes to regulate transcription events loosens up the chromatin to permit the recruitment of key repair players including XPC [97].

### 3.2. CSNK2A1 (CK2*α*1) Signaling Pathway Regulating NER

Casein kinase 2 (CSNK2A1) is a serine/threonine kinase involved in various signaling pathways and has the potential to modulate NER's activity. Recent studies demonstrated that CSNK2A1 could be a potential regulator of the single- and double-stranded breaks of the DNA [98, 99]. X-ray repair cross-complementing protein 1 (XRCC1), a DNA repair protein playing a critical role in the NER particularly at the level of the DNA synthesis and ligation step in the NER, appeared to be a target for CSNK2A. The latter improves the stability of the XRCC1-ligase III complex by triggering phosphorylation of XRCC1 which positively influences NER's activity [99, 100]. Also, XPC appears to be another target for CSNK2A1 by which phosphorylation can occur on the amino acid serine at position 94, thus enhancing photoproducts repair at the level of the NER [101].

### 3.3. MAPK Signaling Pathway Regulating NER

The mitogen-activated protein kinase (MAPK) pathway is responsible for various cellular processes. This pathway comprises different signaling cascades of which the Ras-Raf-Mek-extracellular signal-regulated kinase 1 and 2 (ERK1/2) is responsible for conveying the signal into the nucleus upon RTK activation. The standard pathway begins upon the coupling of epidermal growth factor (EGF) on its receptor inducing receptor dimerization and phosphorylation of tyrosine residues in the cytosolic receptor, then binding of GRB2 which is an adaptor protein and SOS, the Ras guanine nucleotide exchange factor located near Ras protein. SOS exchanges GDP bound to Ras by GTP bound to Ras making it active. Activated Ras binds and turns on Raf (serine/threonine kinase) which in turn activates MEK having dual activity as a serine/threonine and tyrosine kinase activates MAPK (JNK, ERK1/2, or MAPK14) that dimerizes and translocates to the nucleus to activate ternary complex factor (TCF) that enables further activation of transcription factors. Besides, MAPK14 is responsible for halting the cell cycle progression as well as regulating programmed cell death, while MAPK1 is responsible for controlling cellular proliferation and differentiation processes [102]. Activation of ERK1/2 has been shown to decrease lesions [103]. Upon UVR exposure, EP300 is enlisted to the harm site in the heterochromatin and is phosphorylated by MAPK14, MAPK1, and AKT1 expanding histone acetyltransferase (HAT) action to add acetyl groups to H3 and H4 histones prompting chromatin unwinding thus favoring the recruitment of XPC, DDB2, and other players of the NER pathway [104].

### 3.4. NFE2L2 (NRF2) Signaling Pathway Regulating NER

Nuclear factor erythroid 2-related factor 2 (NRF2), also known as nuclear factor erythroid-derived 2-like 2, is a transcription factor classified as a basic leucine zipper (bZIP) responsible for controlling the expression of genes exhibiting antioxidant activities (such as the NFE2L2 gene) to protect against oxidative attacks causing inflammation and lesions [105]. Oxidation of lipids, caused by oxidative damage can block the activity of NER in terms of DNA photolesion repair [106]. Furthermore, the inflammation-inferred monochloramine (NH2CL) represses NER by means of the restraint of p53's phosphorylation [107]. It was additionally shown that the exposure of epithelial cells to H_2_O_2_ diminishes the NER ability to less than half of its total activity (<50%) [108]. In this manner, since NER is repressed by oxidative stress, antioxidants can help forestall such restraint. In oxidative stress conditions, NFE2L2 will be translocated to the nucleus where it interacts with musculoaponeurotic fibrosarcoma (MAF) transcription factors and bind to antioxidant-responsive element (ARE) present within the DNA sequence [109]. Transcription of this region will encode for various important antioxidant enzymes (such as glutathione S-transferase (GST), catalase, peroxidase, and superoxide dismutase) which positively influence NER's activity [110–112].

## 4. XPC and Signaling Pathways

**Figure 4:**
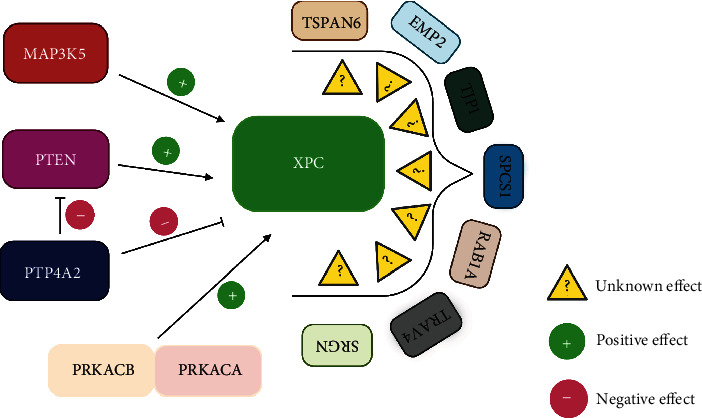
Proteins related to signaling pathways regulating XPC's activity. MAP3K5, PTEN, PRKACB, and PRKACA appeared to have a positive effect, while for PTP4A2 showed a negative effect on XPC's activity. SRGN, TRGV4, RAB1A, SPCS1, TJP1, EMP2, and TSPAN6 are newly identified interactors but with unknown effect on XPC's activity. “Created with http://BioRender.com/.”

XPC is a key player of the GG-NER pathway involved in sensing helical distortions formed upon DNA damage. XPC is formed of around 940 amino acids and possess various domains involved in the interaction with the damaged parts of the DNA as well as other repair factors to proceed in the repairing process [113]. Although the role of XPC in signaling pathways is not yet well understood, a recent study has demonstrated the association between XPC and proteins playing important roles in signaling pathways using the yeast two-hybrid system technique [114]. XPC can be regulated by the MAPK family, specifically p38 MAPK which is required for the recruitment of XPC and TFIIH to the harmed DNA locales [115]. Mitogen-activated protein kinase kinase kinase 5 (MAP3K5) who is responsible for the initiation of the p38 pathway has been identified as an interactor with XPC, thus acting as a positive regulator. Furthermore, PTEN also appeared to positively influence XPC at the transcriptional level [116]. Protein tyrosine phosphatase type IVA, part 2 (PTP4A2), otherwise called PRL2, is a phosphatase having an oncogenic potential and acting as a repressor for PTEN leading to its downregulation, thus allowing the further progression of the AKT signaling pathway. Taken all together, inhibiting PTEN would negatively influence XPC at the transcriptional level, thus affecting DNA damage recognition in GG-NER. Cyclic adenosine monophosphate-dependent protein kinase A (cAMP-dependent PKA) is a member of the protein kinases family that has quite essential roles at the cellular levels like glucose, lipid, and glycogen metabolism. PKA comprises 4 subunits: two regulatory and two catalytic subunits present in all of its variants. XPC was shown to associate with two variants of the catalytic subunit of PKA—PRKACA and PRKACB belonging to the PKA pathway. PRKACB or PRKACA could exert their effects on the GG-NER by either driving XPC to the nucleus or acting at the chromatin level facilitating remodeling events. Other proteins related to signaling pathways include serglycin (SRGN), T-cell receptor gamma variable 4 (TRGV4), member RAS oncogene family (RAB1A), signal peptidase complex subunit 1 (SPCS1), tight junction protein 1 (TJP1), epithelial membrane protein 2 (EMP2), and tetraspanin 6 (TSPAN6) appeared also to associate with XPC [114] (Figure 4). SRGN and TRGV4 play quite important roles in immune responses, TJP1 is implicated in the formation of tight junctions as well as migration processes, RAB1A has roles in proteins and vesicles trafficking between the endoplasmic reticulum and the Golgi apparatus, TSPAN6 has been found to act as an activator of the NF-*κ*B pathway [117], SPCS1 a transmembrane protein in the endoplasmic reticulum, and EMP2 has roles in blastocyst implantation [118]. Although these proteins are identified as interactors with XPC, their impact on XPC's role are unrevealed yet.

## 5. XPC and Skin Cancers

XP is caused by mutations in genes involved in the NER pathway, and its naming comes from the genes which are mutated (XP-A to XP-G and XP-V). Patients acquiring XP have a risk to develop skin cancers 10,000-fold more than normal patients because of their high sensitivity to UVR. For both XP and non-XP having skin cancers, dipyrimidine sites appear to be the most targeted region where mutations occur. For non-XP tumors, mutations are mostly C to T transitions while for XP tumors they are basically CC to TT pair mutations. In addition to that, the most important distinction between NMSC from XP and non-XP patients is their number and the time of appearance. While non-XP NMSC occurs when the patients are generally at late stages of life (50–60 years), XP NMSC appears at early stages of life (3–5 years old) and with a higher incidence rate [119]. Out of XP cases, XPC patients are characterized by having a nonfunctional global genome repair but a normally functional transcription coupled repair because XPC is not required for the TC-NER subpathway. UVB-inducing mutations in the nonexpressed regions of the whole genome would then lead to neoplastic transformation [120]. XPC knockout in mice has been shown to favor the development of spontaneous as well as UV-induced skin cancers [121, 122]. A recent study demonstrated that the downregulation of XPC favors the reprograming of the cellular metabolic processes thus allowing the generation of ROS via NADPH oxidase-1 (NOX1). When activated, the dissociated subunits of NOX1 will merge forming an active enzymatic complex to produce superoxide (O_2_-) from O_2_, requiring NADPH as a substrate [123]. Besides, the rise in the levels of ROS might cause mutations in tumor suppressor genes inactivating them as well as activating mutations in oncogenes, where normal cell cycle processes become disrupted and these cells could evade programmed cell death, and finally progresses to develop cancer cells. NOX-induced ROS production could lead to the activation of the PI3k/AKT as well as kinases responsible for survival responses like Src and transcription factors (NF-*κ*B) and could act as an inhibitor of PTEN and other phosphatases which are responsible for regulating negatively the PI3K/AKT pathway. Thus, XPC loss-driving metabolic disruption could be a key for SCC development [124, 125]. The p53 protein can affect the function of DNA repair systems especially NER either directly or indirectly. A recent study had generated XPC (-/-) p53(-/-) mutant mice models and recognized an elevated exacerbation in UVB-induced keratosis and accelerated skin cancer appearance compared with mice that are only XPC(-/-) homozygous mutants having a wild-type p53(+/+) [126, 127]. Keratosis is a precancerous condition that could rise into SCCs. Normal patients having skin cancers have around 50% of p53 mutations while this percentage rises to 90% in patients having skin cancer and XP disease including XPC. This increase in mutations is attributed to UVB exposure where UV signatures impact the TP53 gene which can be a hallmark favoring skin cancer progression. P16INK4a and ARF are classified as two tumor suppressor proteins, encoded by INK4a/ARF locus, which works together to regulate various cell cycle processes including the p53 and RB pathways. Furthermore, a recent study developed mouse strains lacking XPC (-/-) and INK4a/ARF (-/-) and were subjected to UVB. The results had shown a quite significant elevation in the stimulation of epithelioid cell melanomas in XPC (-/-) and INK4a/ARF (-/-) mice when being compared with XPC (-/-) and INK4a/ARF (+/+) mice [128]. Mutations induced by UV appeared in PTCH and p53 genes in XP and non-XP patients having BCC [129]. Thus, deciphering the dysregulated signaling pathways in XPC patients will draw a correlation on how skin cancers develop in these patients.

## 6. Conclusion

Till date, XPC patients still lack an effective treatment and follow preventative strategies. The setting up of a new disease-modeling strategy of the highly prone skin cancers, xeroderma pigmentosum, using the patient-derived induced pluripotent stem cells (iPSCs) will allow us to understand skin cancer etiology, to identify risk factors in individuals, to discover protein biomarkers for the initiation, progression, and invasion of skin cancer, and finally, to design novel therapeutic or prevention strategies. This strategy will have several outcomes situated at different levels, like understanding the difference in the etiology of UVR-induced BCC, SCC, and melanomas in order to establish an effective prevention strategy, deciphering the relationship between genotoxicity, DNA repair capacity, and cancer incidence. The discovery of skin “tumorgenesis-initiation” biomarkers or markers for high risk (such as DNA photoproducts, cytokines, and inflammatory factors) will allow a better identification of people at risk and improvement of preventive interventions in order to limit the increase in skin cancer incidence. Note that 20,000 Europeans die every year from skin cancer and at least 100-200 times more are treated for skin cancer, mostly by surgery. So, skin cancer poses considerable burden on patients and health care services worldwide and the situation will only get worse with the aging of the population. Targeting the dysregulated signaling pathways at the molecular level might be quite promising to decrease the photosensitivity as well as preventing skin cancer occurrence in XPC patients. In this review, we focused on the signaling pathways dysregulated in NMSC and CM, signaling pathways modulating NER's function, proteins linked to signaling pathways affecting XPC's activity, and little information present about XPC's loss and skin cancers. It is worth mentioning that gene therapy could be also quite promising to replace the XPC mutant gene by a wild-type version, but this technology requires a lot of optimization.
